# Biodiversity of *Aspergillus* section *Flavi* in Europe in relation to the management of aflatoxin risk

**DOI:** 10.3389/fmicb.2014.00377

**Published:** 2014-07-21

**Authors:** Giancarlo Perrone, Antonia Gallo, Antonio F. Logrieco

**Affiliations:** ^1^Institute of Sciences of Food Production, National Research CouncilBari, Italy; ^2^Unit of Lecce, Institute of Sciences of Food Production, National Research CouncilLecce, Italy

**Keywords:** *Aspergillus*, aflatoxin, genetic variation, climate change, atoxigenic strains, biocontrol

## Abstract

Aflatoxins and the producing fungi *Aspergillus* section *Flavi* are widely known as the most serious and dangerous mycotoxin issue in agricultural products. In Europe, before the outbreak of aflatoxins on maize (2003–2004) due to new climatic conditions, their contamination was confined to imported foods. Little information is available on molecular biodiversity and population structure of *Aspergillus* section *Flavi* in Europe. Preliminary reports evidenced the massive presence of *Aspergillus flavus* L -morphotype as the predominant species in maize field, no evidence of the highly toxigenic S-morphotype and of other aflatoxigenic species are reported. The risk of a shift in traditional occurrence areas for aflatoxins is expected in the world and in particular in South East of Europe due to the increasing average temperatures. Biological control of aflatoxin risk in the field by atoxigenic strains of *A. flavus* starts to be widely used in Africa and USA. Studies are necessary on the variation of aflatoxin production in populations of *A. flavus* to characterize stable atoxigenic *A. flavus* strains. The aim of present article is to give an overview on biodiversity and genetic variation of *Aspergillus* section *Flavi* in Europe in relation to the management of aflatoxins risk in the field.

## INTRODUCTION

The relatively recent outbreak of aflatoxins risk contamination in Europe has driven the EU researchers to investigate on this topic usually confined to tropical and sub-tropical areas. In this respect, aflatoxins and aflatoxigenic fungi are by far the most serious, dangerous and studied mycotoxin issue in agricultural products.

Aflatoxins are secondary metabolites synthesized by several *Aspergillus* species and are highly toxic to humans and animals when ingested at high concentrations. They may cause severe liver damage accompanied by jaundice, hepatitis and death, especially in developing countries (Probst et al., 2007). In addition, they are genotoxic, carcinogenic, and teratogenic for both humans and animals. Daily consumption of foods contaminated with low levels of aflatoxin B_1_ (AFB_1_) can result in chronic aflatoxicosis with stunting in children, immune suppression, cancer, and reduced life expectancy (Shephard, 2008).

Aflatoxins could extensively contaminate pre-harvest corn, cotton, soybean, peanuts, and tree nuts, and in addition residues from contaminated feed may appear in milk (Bennett and Klich, 2003).

In general, and in most of the published studies, *A. flavus* and *A. parasiticus* are the major prominent species associated in aflatoxin contamination of agricultural crops. However, recently additional species of Section *Flavi* have been reported to be responsible of aflatoxin contamination, i.e., *A. arachidicola* in peanuts and *A. nomius* in corn, nuts, and brazil nuts, especially in certain geographical area (Varga et al., 2011). New aflatoxigenic species *A. mottae*, *A. sergii* and *A. transmontanensis* have been described from maize and almonds in Portugal, they all belong to *A. parasiticus* group (Soares et al., 2012). *A. flavus* isolates produce AFB_1_ and AFB_2_ and often cyclopiazonic acid (CPA), while most *A. parasiticus* strains produce AFG_1_ and AFG_2_ in addition to AFB_1_ and AFB_2_ but never produce CPA (Horn and Dorner, 1999). Production in *A. flavus* is highly variable and depends on genotype, substrate and geographic origin, climate change and agronomic practice. Moreover, *A. flavus* is considered the predominant contaminating organism in soil and in agricultural areas and this species is more invasive and out competes *A. parasiticus* when both species are together in soil. So, most of the extensive researches have been conducted on this species and on its diverse assemblage of strains: different vegetative compatibility groups (VCGs), sclerotial type variants, toxigenic and atoxigenic strains (Ehrlich, 2014). In fact, *A. flavus* populations include isolates with two morphologically distinct sclerotial size variants, L strains with average sclerotial size >400 μm and S strains with sclerotial size <400 μm. Both these morphotypes are found in maize regions around the world; L strains are usually less toxigenic than S strains and produce only AFBs, while S strains are often high producer of aflatoxins and could be divided in two chemotypes: the S_BG_ producing both AFBs and AFGs, and the S_B_ producing only AFBs. The closely related new species *A. parvisclerotigenus* and *A. minisclerotigenes* were potentially associated to the S_BG_ and S_B_ taxons of *A. flavus*, respectively (Saito and Tsuruta, 1993; Pildain et al., 2008). Isolates that share morphological characters with S_BG_ have been reported from Thailand, Argentina, and Australia, but the exact taxonomic designation of S_BG_ remains unclear (Cotty and Cardwell, 1999; Donner et al., 2009). More recently, Probst et al. (2012) assigned four phylogenetic distinct groups to the S strains: one seems to belong to *A. minisclerotigenes* (S_BG_ from Australia, Nigeria, Argentina, USA); the second is the highly toxic Kenyan S_B_ group (Probst et al., 2007), and a third is the S_B_ group from elsewhere in the world, e.g., Thailand, United States, and Philippines. Finally, there is the S_BG_ strain group from Nigeria. In general, members of the S_BG_ group are found in locations where high levels of aflatoxin contamination occur.

It is important to underline that, based on actual surveys, the Europe population of *A. flavus* seems constituted only by L strains, none of the studies conducted have found *A. flavus* S strains. In Europe, the occurrence of *A. parasiticus* in maize seems to be very rare, while it resulted more distributed in almond especially in Portugal with an atypical chemotype producing only AFBs toxins (Rodrigues et al., 2011).

Incidence of aflatoxin outbreaks are most severe in tropical and subtropical areas around the world and also temperate regions, such as the United States Midwest are subject to occurrence of aflatoxin contamination. Until 2004, the European perspective regarding aflatoxin contamination was confined to imported foods such as peanut cake, palm kernel, copra, and corn gluten meal (depending of origin; European Food Safety Authority [EFSA], 2004). Several surveys have been conducted for detection of AFs in feed samples in Europe founding a small percentage of materials contaminated with AFB_1_ concentration above the regulatory limit. In fact, a survey of 110 maize samples in Northern Italy in 2003, initially planned to monitor the occurrence of fumonisins, showed 75% positive samples to AFBs with a mean of 4.4 and a maximum of 154.5 μg/kg (Piva et al., 2006). In 2006, aflatoxin contaminated rice meal used in dairy cattle feed production had been identified as the cause of elevated AFM_1_ levels in Swedish milk.

However, a big survey conducted by European Food Safety Authority [EFSA] (2007) evidenced the emerging issue of potential aflatoxin contamination of corn, almonds, pistachios grown in areas of Southern Europe, due to the subtropical climate occurring in some recent years. In the study of Ibáñez-Vea et al. (2012), detectable levels of AFB_1_ were reported for all the 123 Spanish barley samples from 2007 to 2008, and it was found to co-occur with other mycotoxins like ochratoxin A and zearalenone. In the recent work of Streit et al. (2013), samples of feed and feed raw materials from over the world were analyzed during an 8 year period for contamination with different mycotoxins. Regarding aflatoxin contamination, European samples originated primarily from Central Europe showed a high percentage of around 30% positive samples, albeit the pool of samples tested was made up by finished feeds or imported feedstuffs for more than half. A recent warning for maize contamination in Northern Italy was issued in 2012–2013 in consequence of drought conditions favorable to *A. flavus* infection (Andreotti, 2013; Causin, 2013).

In the following chapters of this mini-review we intend to analyze the most important critical points that should be studied and keep under audit to prevent and reduce the spreading of aflatoxin risk in Europe in the next decades.

## CLIMATE CHANGE AND RISK OF AFLATOXIN CONTAMINATION IN EUROPE

A wide body of evidence demonstrates that the ability of fungi to grow, survive and interact with a large variety of crop species and to produce mycotoxins is greatly influenced by environmental factors, mainly temperature, relative humidity, insects. These factors are greatly related to climate change and to the variation of temperature and rainfalls. In this sense, food safety has become a very important issue worldwide and the potential effects of climate change on yields and quality of food crops, especially for mycotoxins, have received special attention in the last years, in particular from a risk analysis perspective (Miraglia et al., 2009; Magan et al., 2011). A shift in traditional occurrence areas for mycotoxins is to be expected due to the increasing average temperatures. In this respect, the Mediterranean zones have been identified as a climate change hotspot where extreme changes in temperature, CO_2_ levels and rainfall patterns are predicted. Regarding aflatoxins, their contamination events are more prevalent during times of high heat and drought, which may stress the host plant thereby facilitating *A. flavus* infection (Schmidt-Heydt et al., 2009; Mohale et al., 2013).

In 2003 and sporadically in the following years a hot and drying season led to severe *A. flavus* infection of maize in Northern Italy, as mentioned above (Piva et al., 2006; Battilani et al., 2008). The use of this maize as feedstuff for dairy cattle led to a widespread AFM_1_ contamination in milk and several thousands of tons of milk exceeding the EU legal limit of 0.05 μg/kg had to be discarded. In the 2 years following this incidence from the study of Decastelli et al. (2007) the presence of AFM_1_ in milk and AFB_1_ in feed was higher than the maximum allowable in 1.7% of raw milk samples and in 8.1% of feed samples. In 2005, the presence of these aflatoxins was below the limits of EU regulations. So, because of the very dry conditions in those years, *A. flavus* became a significant problem.

Under heat/drought stress also peanuts and pistachio can develop cracking in pods or hull splitting resulting in a significant increase in aflatoxin contamination (Cotty and Jaime-Garcia, 2007). Drought is also a major stress for the plants and undermines their natural immunity against pathogens like mycotoxin-producing fungi (Bircan et al., 2008; Kebede et al., 2012).

New strategies to monitor and predict mycotoxin contamination, either in specific foods or in geographical regions are of recent development, they could be useful in the next years for identify and predict environmental conditions present in regions that may favor mycotoxin proliferation. In this regard, some models have been created to predict aflatoxin for pistachio nuts (Marín et al., 2012), peanuts (Boken et al., 2008), and other crops (Masuoka et al., 2010). More recently, as in Northern Europe *A. flavus* became a dominant pathogen in maize, Battilani et al. (2013) have developed a mechanistic model “AFLA-maize” for the prediction of *A. flavus* infection and AFB_1_ contamination in this crop.

Another potential consequence of climate change is that the biocontrol strain could be an inadvertent cause of increased damage to the plant, especially if growing conditions are less favorable for cultivation. Changes in soil environment and its microbiome due to temperature increase, could also subject the crop to amplified damage. So, across Europe, it is relevant to improve harmonization of surveillance and monitoring of aflatoxins; improve database on the geographical distribution and prevention methods for aflatoxin; develop models for the prediction of aflatoxin contamination in the new biogeographical agricultural scenarios.

## BIODIVERSITY AND GENETIC DIVERSITY OF *Aspergillus* SECTION *Flavi* IN EUROPE

Soil populations of *A. flavus* are typically composed of isolates from hundreds of different VCGs which reflect phenotypic differences (or similarity) among individuals (Leslie, 1993). Individuals (genotypes) of a fungal species having the same heterokaryon or vegetative incompatibility loci can fuse and undergo genetic exchange through parasexuality (Glass et al., 2000). Fungal isolates that form stable heterokaryons are considered to belong to same VCGs. In *A. flavus* populations, most of variations in morphology and mycotoxin production can be attributed to differences among VCGs. Vegetative compatibility group was believed to be a strong barrier to genetic exchange but recent studies found that VCGs are able to outcross, leading to new VCGs and thereby increased diversity (Olarte et al., 2012).

Recently, sexual reproduction was demonstrated in *A. flavus* which resulted to be an heterothallic fungus with two mating type loci, MAT1-1 and MAT1-2 maintained separately in homokaryotic isolates (Ramirez-Prado et al., 2008; Horn et al., 2009). Recombination can occur within conidia or sclerotia when they harbor multiple nuclei of different mating type and thereby capable of recombination.

Sexual recombination occurs in *A. flavus* through the meiotic process of independent assortment and crossing over that may influence the toxin phenotype of *A. flavus* strains, with a reduction or a complete loss of toxicity (Olarte et al., 2012). The majority of the genetic variation in mycotoxin production arises from mutations in the aflatoxin biosynthetic gene cluster (Chang et al., 2005, 2006), including gene loss, recombination, DNA inversions, partial deletions, translocations, and other genomic rearrangements of the cluster likely due to proximity of the cluster to the telomeric region of chromosome (Carbone et al., 2007).

Comparative analyses of the aflatoxin cluster in various *Aspergillus* species have underlined the complex evolutionary history of this cluster and its role in species adaptation and diversification (Ehrlich et al., 2003; Moore et al., 2009). From analyses of *Aspergillus* populations, several distinct deletions within aflatoxin cluster have been described that may each be responsible for atoxigenicity in various isolates. Either part or the entire biosynthetic cluster resulted deleted, or the non-aflatoxigenicity was associated to inability to amplify selected aflatoxin genes (Chang et al., 2005; Criseo et al., 2008; Donner et al., 2010).

Not many studies on the molecular diversity of *Aspergillus* populations isolated in Europe are available. Among these, the work of Gallo et al. (2012) was about an *A. flavus* population isolated from maize in 2003, during the first outbreak of aflatoxin contamination documented in Northern Italy (Piva et al., 2006; Giorni et al., 2007). The strains were analyzed for the presence of seven aflatoxin biosynthesis genes, including the regulatory genes *aflR* and *aflS*, in relation to their capability to produce AFB_1_. All aflatoxin producing isolates exhibited the complete set of amplification products, whereas non-producing isolates did not yield amplified products for three, four or all seven tested genes. The genetic diversity of *A. flavus* populations collected from maize kernels in Northern Italy from 2003 to 2010 was assessed by analysis of VCG and presence or absence of several aflatoxin genes by Mauro et al. (2013). Forty-eight VCGs were identified by means of complementation between nitrate non-utilizing mutants. Twenty-five of these VCGs contained only atoxigenic isolates and the remaining 23 only aflatoxin producers. In addition six deletion patterns of genes in aflatoxin cluster were detected. Regarding the atoxigenic isolates, 12 of them had no deletion in the cluster, 10 had the entire cluster deleted and only one had a deletion pattern only seen once before in Nigeria, with only two genes amplified out of the thirteen tested.

The genetic variability of aflatoxin cluster in non-aflatoxigenic isolates appears diversified and complex but its understanding is important for the selection of safe and effective non-producing strains potentially usable in biocontrol for limiting aflatoxin contamination.

## ATOXIGENIC STRAIN AND GENETIC VARIATION IN AFLATOXIN CONTROL STRATEGY

Interest in the variation of aflatoxin production by strains of *Aspergillus* section *Flavi* has increased recently because atoxigenic strains of *A. flavus* are being used as biological control agents to reduce the risk of aflatoxin contamination (Atehnkeng et al., 2008; Wu and Khlangwiset, 2010). Atoxigenic strains may displace wild-type aflatoxigenic strains in crop environments so only the non-aflatoxigenic population of fungi would be present in the field.

The effectiveness of pre-harvest biocontrol strategies using atoxigenic strains is based on competition for substrate, the potential production of inhibitory metabolites, and on their inability to recombine with native toxigenic strains, thus preventing the reacquisition of aflatoxigenicity (Ehrlich and Cotty, 2004; Abbas et al., 2011). Anyway, the choice of a candidate biocontrol atoxigenic *A. flavus* strain could not be based only on the phenotypic characteristic of atoxigenicity; it is necessary to investigate the genotypic condition. The long-term effect of atoxigenic biocontrol strains on native population depends on possibility of sexual recombination, in presence of which the high aflatoxin heritability will induce the regaining of ability to produce aflatoxins. There is never complete inheritance of the atoxigenic phenotype in the offspring of a biocontrol parent, so the use of atoxigenic strains biocontrol with lacking cluster genes would be preferable to one with intact biosynthetic cluster (Olarte et al., 2012; Moore, 2014).

Other factors should be considered for a successful application of biocontrol strategy such as the better understanding of natural diversity of *A. flavus* populations in agricultural soil, the ability of the introduced non-aflatoxigenic strains to recombine with the existing aflatoxigenic strains, the adaptation of *A. flavus* isolates for growth on the plant, the potential damage to the plant from the introduced strain, the potential effect on the soil microenvironment, the timing and the economical cost of application of biocontrol isolates, the potential production of other toxic metabolites, in addition to aflatoxins, which could affect animal health (Ehrlich, 2014).

This form of competitive exclusion of toxigenic strains by non-aflatoxigenic biocontrol strains has been demonstrated under field conditions in cotton (Cotty, 1994), peanuts (Dorner, 2005; Alaniz Zanon et al., 2013) and maize (Abbas et al., 2006).

The aflatoxigenic isolate AF36, which is unable to produce aflatoxin because of a point mutation in the polyketide synthase gene (*pksA*) necessary for aflatoxin biosynthesis (Ehrlich and Cotty, 2004), is registered to be used in USA for the management of aflatoxin contamination in cottonseed fields. The strain NRRL21882 is the active ingredient of Afla-Guard, a biocontrol formulation consisting of spore-coated barley seeds and used in peanut fields (Chang and Hua, 2007); the isolate is missing the entire aflatoxin and CPA gene clusters and therefore is unable to produce both mycotoxins (Chang et al., 2005, 2009). Recently the possibility to replace grain seeds in this formulation with bioplastic based granules has been explored in field experiments conducted in Northern Italy showing that bioplastic formulations are effective in reducing aflatoxin contamination in corn (Accinelli et al., 2014). In some cases the competing fungi are used as cocktails that include application of multiple strains of non-aflatoxigenic *A. flavus* (Wu et al., 2013).

## CONCLUSION

The occurrence of AFB_1_ at high levels in Europe in the years 2003–2004 and 2012–2013 underlines the fact that the climate change will entail a change in the mycotoxin distribution patterns observed today. Global trade of plant products can also contribute to the spread of aflatoxigenic fungi and to the increase of diversity of local fungal populations. The study of diversity of aflatoxigenic fungi occurring in maize in Europe, under different points of view-morphological, molecular, metabolic, and plant pathological is essential for the development of strategies for the control of aflatoxin contamination. In this regard, the molecular characterization of native atoxigenic strains, acting through competitive exclusion of aflatoxin producers, with superior adaptation to a geographical region, should provide benefit of long-term displacement of toxigenic strains in maize environment. Additional information on the behavior of these atoxigenic isolates in the target agro-ecosystem will be needed to choose the best biological control agents. Finally, the development of predictive models for aflatoxins occurrence based on regional weather data would be a valuable tool to estimate the risk of contamination after a given growing season, together with using biopesticides in the frame of an integrated pest management (IPM).

## Conflict of Interest Statement

The authors declare that the research was conducted in the absence of any commercial or financial relationships that could be construed as a potential conflict of interest.

## References

[B1] Abbas H. K. Zablotowicz R. M. Abel C. A. (2006). Biocontrol of aflatoxin in corn by inoculation with non-aflatoxigenic *Aspergillus flavus* isolates. *Biocontrol. Sci. Techn.* 16 437–449 10.1080/09583150500532477

[B2] Abbas H. K. Zablotowicz R. M. Horn B. W. Phillips N. A. Johnson B. J. Jin X. (2011). Comparison of major biocontrol strains of non-aflatoxigenic *Aspergillus flavus* for the reduction of aflatoxins and cyclopiazonic acid in maize. *Food Addit. Contam.* 28 198–208 10.1080/19440049.2010.544680 21259141

[B3] Accinelli C. Abbas H. K. Vicari A. Shier W. T. (2014). Aflatoxin contamination of corn under different agro-environmental conditions and biocontrol applications. *Crop Prot.* 63 9–14 10.1016/j.cropro.2014.04.021

[B4] Alaniz Zanon M. S. Chiotta M. L. Giaj-Merlera G. Barros G. Chulze S. (2013). Evaluation of potential biocontrol agent for aflatoxin in Argentinean peanuts. *Int. J. Food Microbiol.* 162 220–225 10.1016/j.ijfoodmicro.2013.01.017 23454811

[B5] Andreotti L. (2013). Torna la minaccia aflatossine per il mais 2013. *L’Informatore Agrario* 32 15

[B6] Atehnkeng J. Ojiambo P. S. Ikotun T. Sikora R. A. Cotty P. J. Bandyopadhyay R. (2008). Evaluation of atoxigenic isolates of *Aspergillus flavus* as potential biocontrol agents for aflatoxin in maize. *Food Addit. Contam.* 25 1264–1271 10.1080/02652030802112635 18608502

[B7] Battilani P. Camardo Leggieri M. Rossi V. Giorni P. (2013). AFLA-maize, a mechanistic model for *Aspergillus flavus* infection and aflatoxin B1 contamination in maize. *Comput. Electron. Agric.* 94 38–46 10.1016/j.compag.2013.03.005

[B8] Battilani P. Pietri A. Barbano C. Scandolara A. Bertuzzi T. Marocco A. (2008). Logistic regression modeling of cropping systems to predict fumonisin contamination in maize. *J. Agric. Food Chem.* 56 10433–10438 10.1021/jf801809d 18841987

[B9] Bennett J. W. Klich M. A. (2003). Mycotoxins. *Clin. Microbiol. Rev.* 16 497–516 10.1128/CMR.16.3.497-516.2003 12857779 PMC164220

[B10] Bircan C. Barringer S. A. Ulken U. Pehlivan R. (2008). Increased aflatoxin contamination of dried figs in a drought year. *Food Addit. Contam.* 25 1400–1408 10.1080/02652030802163414 19680848

[B11] Boken V. K. Hoogenboom G. Williams J. H. Diarra B. Dione S. Easson G. L. (2008). Monitoring peanut contamination in Mali (Africa) using the AVHRR satellite data and a crop simulation model. *Int. J. Remote Sen.* 29 117–129 10.1080/01431160701264250

[B12] Carbone I. Ramirez-Prado J. H. Jakobek J. L. Horn B. W. (2007). Gene duplication, modularity and adaptation in the evolution of the aflatoxin gene cluster. *BMC Evol. Biol.* 7:111 10.1186/1471-2148-7-111 PMC1949824 17620135

[B13] Causin R. (2013). Cosa sapere per prevenire il rischio aflatossine nel mais. *L’Informatore Agrario* 3 46–49

[B14] Chang P. K. Ehrlich K. C. Hua S. S. (2006). Cladal relatedness among Aspergillus oryzae isolates and *Aspergillus flavus* S and L morphotypes. *Int. J. Food Microbiol.* 108 172–177 10.1016/j.ijfoodmicro.2005.11.008 16430983

[B15] Chang P. K. Horn B. W. Dorner J. W. (2005). Sequence breakpoints in the aflatoxin biosynthesis gene cluster and flanking regions in nonaflatoxigenic *Aspergillus flavus* isolates. *Fungal Genet. Biol.* 42 914–923 10.1016/j.fgb.2005.07.004 16154781

[B16] Chang P. K. Horn B. W. Dorner J. W. (2009). Clustered genes involved in cyclopiazonic acid production are next to the aflatoxin biosynthesis gene cluster in *Aspergillus flavus*. *Fungal Genet. Biol.* 46 176–182 10.1016/j.fgb.2008.11.002 19038354

[B17] Chang P. K. Hua S. S. (2007). Nonaflatoxigenic *Aspergillus flavus* TX9-8 competitively prevents aflatoxin accumulation by A. flavus isolates of large and small sclerotial morphotypes. *Int. J. Food Microbiol.* 114 275–279 10.1016/j.ijfoodmicro.2006.09.017 17140692

[B18] Cotty P. J. (1994). Influence of field application of an atoxigenic strain of *Aspergillus flavus* on the populations of *A. flavus* infecting cottonbolls and on the aflatoxin content of cottonseed. *Phytopathology* 84 1270–1277 10.1094/Phyto-84-1270

[B19] Cotty P. J. Cardwell K. F. (1999). Divergence of West African and North American communities of *Aspergillus* section *Flavi*. *Appl. Environ. Microb.* 65 2264–2266 10.1128/aem.65.5.2264-2266.1999 PMC91331 10224034

[B20] Cotty P. J. Jaime-Garcia R. (2007). Influences of climate on aflatoxin producing fungi and aflatoxin contamination. *Int. J. Food Microbiol.* 119 109–115 10.1016/j.ijfoodmicro.2007.07.060 17881074

[B21] Criseo G. Racco C. Romeo O. (2008). High genetic variability in non-aflatoxigenic *Aspergillus flavus* strains by using Quadruplex PCR-based assay. *Int. J. Food Microbiol.* 125 341–343 10.1016/j.ijfoodmicro.2008.04.020 18538431

[B22] Decastelli L. Lai J. Gramaglia M. Monaco A. Nachtmann C. Oldano F. (2007). Aflatoxins occurrence in milk and feed in Northern Italy during 2004–2005. *Food Control* 18 1263–1266 10.1016/j.foodcont.2006.08.006

[B23] Donner M. Atehnkeng J. Sikora R. A. Bandyopadhyay R. Cotty P. J. (2009). Distribution of *Aspergillus* section *Flavi* in soils of maize fields in three agroecological zones of Nigeria. *Soil Biol. Biochem.* 41 37–44 10.1016/j.soilbio.2008.09.013

[B24] Donner M. Atehnkeng J. Sikora R. A. Bandyopadhyay R. Cotty P. J. (2010). Molecular characterization of atoxigenic strains for biological control of aflatoxins in Nigeria. *Food Addit. Contam.* 27 576–590 10.1080/19440040903551954 20455156

[B25] Dorner J. W. (2005). “Biological control of aflatoxin crop contamination,” in *Aflatoxin and Food Safety* ed. Abbas H. K. (New York, NY: Taylor & Francis) 333–352 10.1201/9781420028171.ch16

[B26] Ehrlich K. C. (2014). Non-aflatoxigenic *Aspergillus flavus* to prevent aflatoxin contamination in crops: advantages and limitations. *Front. Microbiol.* 5:50 10.3389/fmicb.2014.00050 PMC3918586 24575088

[B27] Ehrlich K. C. Cotty P. J. (2004). An isolate of *Aspergillus flavus* used to reduce aflatoxin contamination in cottonseed has a defective polyketide synthase gene. *Appl. Microbiol. Biotechnol.* 65 473–478 10.1016/S1087-1845(02)00509-501 15235754

[B28] Ehrlich K. C. Montalbano B. G. Cotty P. J. (2003). Sequence comparison of aflR from different *Aspergillus* species provides evidence for variability in regulation of aflatoxin production. *Fungal Genet. Biol.* 38 63–74 10.1016/S1087-1845(02)00509-1 12553937

[B29] European Food Safety Authority [EFSA] (2004). Opinion of the Scientific Panel on Contaminants in the Food Chain on a request from the Commission related to Aflatoxin B1 as undesirable substance in animal feed. *EFSA J.* 39 1–27

[B30] European Food Safety Authority [EFSA] (2007). Opinion of the Scientific Panel on Contaminants in the Food Chain on a request from the Commission related to the potential increase of consumer health risk by a possible increase of the existing maximum levels for aflatoxins in almonds, hazelnuts and pistachios and derived products. *EFSA J.* 446 1–127

[B31] Gallo A. Stea G. Battilani P. Logrieco A. F. Perrone G. (2012). Molecular characterization of an *Aspergillus flavus* population isolated from maize during the first outbreak of aflatoxin contamination in Italy. *Phytopathol. Mediterr.* 51 198–206

[B32] Giorni P. Magan N. Pietri A. Bertuzzi T. Battilani P. (2007). Studies on *Aspergillus* section *Flavi* isolated from maize in northern Italy. *Int. J. Food Microbiol.* 113 330–338 10.1016/j.ijfoodmicro.2006.09.007 17084935

[B33] Glass N. L. Jacobson D. J. Shiu P. K. T. (2000). The genetics of hyphal fusion and vegetative incompatibility in filamentous ascomycete fungi. *Annu. Rev. Genet.* 34 165–186 10.1146/annurev.genet.34.1.165 11092825

[B34] Horn B. W. Dorner J. W. (1999). Regional differences in production of aflatoxin B1 and cyclopiazonic acid by soil isolates of *Aspergillus flavus* along a transect within the United States. *Appl. Environ. Microbiol.* 65 1444–1449 10103234 10.1128/aem.65.4.1444-1449.1999 PMC91204

[B35] Horn B. W. Moore G. G. Carbone I. (2009). Sexual reproduction in *Aspergillus flavus*. *Mycologia* 101 423–429 10.3852/09-011 19537215

[B36] Ibáñez-Vea M. González-Peñas E. Lizarraga E. López de Cerain A. (2012). Co-occurrence of aflatoxins, ochratoxin A and zearalenone in barley from a northern region of Spain. *Food Chem.* 132 35–42 10.1016/j.foodchem.2011.10.023 26434260

[B37] Kebede H. Abbas H. K. Fisher D. K. Bellaloui N. (2012). Relationship between aflatoxin contamination and physiological responses of corn plants under drought and heat stress. *Toxins* 4 1385–1403 10.3390/toxins4111385 23202322 PMC3509714

[B38] Leslie J. F. (1993). Fungal vegetative compatibility. *Annu. Rev. Phytopathol.* 31 127–150 10.1146/annurev.py.31.090193.001015 18643765

[B39] Magan N. Medina A. Aldred D. (2011). Possible climate-change effects on mycotoxin contamination of food crops pre-and postharvest. *Plant Pathol.* 60 150–163 10.1111/j.1365-3059.2010.02412.x

[B40] Marín S. Ramos A. J. Sanchis V. (2012). Modeling *Aspergillus flavus* growth and aflatoxins production in pistachio nuts. *Food Microbiol.* 32 378–388 10.1016/j.fm.2012.07.018 22986204

[B41] Masuoka P. Chamberlin J. Elias M. (2010). Modeling the distribution and probability of aflatoxin occurrence using environmental data. *International Food Policy Research Institute (IFPRI)*. Available at: http://www.ifpri.org/sites/default/files/publications/aflacontrol_wp02.pdf

[B42] Mauro A. Battilani P. Callicott K. A. Giorni P. Pietri A. Cotty P. J. (2013). Structure of an *Aspergillus flavus* population from maize kernels in northern Italy. *Int. J. Food Microbiol.* 162 1–7 10.1016/j.ijfoodmicro.2012.12.021 23340386

[B43] Miraglia M. Marvin H. J. P. Kleter G. A. Battilani P. Brera C. Coni E. (2009). Climate change and food safety: an emerging issue with special focus on Europe. *Food Chem. Toxicol.* 47 1009–1021 10.1016/j.fct.2009.02.005 19353812

[B44] Mohale S. Medina A. Magan N. (2013). Effect of environmental factors on in vitro and in situ interactions between atoxigenic and toxigenic *Aspergillus flavus* strains and control of aflatoxin contamination of maize. *Biocontrol Sci. Technol.* 23 776–793 10.1080/09583157.2013.794895

[B45] Moore G. G. (2014). Sex and recombination in aflatoxigenic *Aspergilli*: global implications. *Front. Microbiol.* 5:32 10.3389/fmicb.2014.00032 PMC3913843 24550903

[B46] Moore G. G. Singh R. Horn B. W. Carbone I. (2009). Recombination and lineage-specific gene loss in the aflatoxin gene cluster of *Aspergillus flavus*. *Mol. Ecol.* 18 4870–4887 10.1111/j.1365-294X.2009.04414.x 19895419

[B47] Olarte R. A. Horn B. W. Dorner J. W. Monacell J. T. Singh R. Stone E. A. (2012). Effect of sexual recombination on population diversity in aflatoxin production by *Aspergillus flavus* and evidence for cryptic heterokaryosis. *Mol. Ecol.* 21 1453–1476 10.1111/j.1365-294X.2011.05398.x 22212063

[B48] Pildain M. B. Vaamonde G. Cabral D. (2008). Analysis of population structure of *Aspergillus flavus* from peanut based on vegetative compatibility, geographic origin, mycotoxin and sclerotia production. *Int. J. Food Microbiol.* 93 31–40 10.1016/j.ijfoodmicro.2003.10.007 15135580

[B49] Piva G. Battilani P. Pietri A. (2006). “Emerging issues in Southern Europe: aflatoxins in Italy,” in *The Mycotoxin Factbook, Food and Feed Topics* eds Barug D. Bhatnagar D. van Egmond H. P. van der Kamp J. W. van Osenbruggen W. A. Visconti A. (Wageningen: Wageningen Academic Publishers) 139–153

[B50] Probst C. Callicot K. A. Cotty P. J. (2012). Deadly strains of Kenyan Aspergillus are distinct from other aflatoxin producers. *Eur. J. Plant Pathol.* 132 419–429 10.1007/s10658-011-9887-y

[B51] Probst C. Njapau H. Cotty P. J. (2007). Outbreak of an acute aflatoxicosis in Kenya in 2004: identification of the causal agent. *Appl. Environ. Microbiol.* 73 2762–2764 10.1128/AEM.02370-2376 17308181 PMC1855601

[B52] Ramirez-Prado J. H. Moore G. G. Horn B. W. Carbone I. (2008). Characterization and population analysis of the mating-type genes in *Aspergillus flavus* and *Aspergillus parasiticus*. *Fungal Genet. Biol.* 45 1292–1299 10.1016/j.fgb.2008.06.007 18652906

[B53] Rodrigues P. Santos C. Venâncio A. Lima N. (2011). Species identification of *Aspergillus* section *Flavi* isolates from Portuguese almonds using phenotypic, including MALDI-TOF ICMS, and molecular approaches. *J. Appl. Microbiol.* 111 877–892 10.1111/j.1365-2672.2011.05116.x 21790915

[B54] Saito M. Tsuruta O. (1993). A new variety of *Aspergillus flavus* from tropical soil in Thailand and its aflatoxin productivity. *Proc. Jpn. Assoc. Mycotoxicol.* 37 31–36

[B55] Schmidt-Heydt M. Abdel-Hadi A. Magan N. Geisen R. (2009). Complex regulation of the aflatoxin biosynthesis gene cluster of *Aspergillus flavus* in relation to various combinations of water activity and temperature. *Int. J. Food Microbiol.* 135 231–237 10.1016/j.ijfoodmicro.2009.07.026 19699547

[B56] Shephard G. S. (2008). Impact of mycotoxins on human health in developing countries. *Food Addit. Contam.* 25 146–151 10.1080/02652030701567442 18286404

[B57] Soares C. Rodrigues P. Peterson S. W. Lima N. Venâncio A. (2012). Three new species of *Aspergillus* section *Flavi* isolated from almonds and maize in Portugal. *Mycologia* 104 682–697 10.3852/11-088 22123651

[B58] Streit E. Naehrer K. Rodrigues I. Schatzmayra G. (2013). Mycotoxin occurrence in feed and feed raw materials worldwide: long-term analysis with special focus on Europe and Asia. *J. Sci. Food Agric.* 93 2892–2899 10.1002/jsfa.6225 23670211

[B59] Varga J. Frisvad J. C. Samson R. A. (2011). Two new aflatoxin producing species, and an overview of *Aspergillus* section *Flavi*. *Stud. Mycol.* 69 57–80 10.3114/sim.2011.69.05 21892243 PMC3161756

[B60] Wu F. Khlangwiset P. (2010). Evaluating the technical feasibility of aflatoxin risk reduction strategies in Africa. *Food Addit. Contam. A* 27 658–676 10.1080/19440041003639582 PMC2882709 20455160

[B61] Wu F. Stacy S. L. Kensler T. W. (2013). Global risk assessment of aflatoxins in maize and peanuts: are regulatory standards adequately protective? *Toxicol. Sci.* 135 251–259 10.1093/toxsci/kft132 23761295 PMC3748761

